# To what extent do sexual hormones influence bipolar disorder?

**DOI:** 10.1192/j.eurpsy.2021.1650

**Published:** 2021-08-13

**Authors:** F. Gonçalves Viegas, F. Ferreira, I. Figueiredo, A. Lourenço

**Affiliations:** Departamento De Saúde Mental, Hospital Prof Dr Fernando Fonseca, Amadora, Portugal

**Keywords:** sexual hormones, oral contraception, mood instability, bipolar disorder

## Abstract

**Introduction:**

It is known that female reproductive events and hormonal treatments can impact the course of bipolar disorder (BD) in women, some of whom are more vulnerable to the development of mood instability under periods of hormonal fluctuation. The mechanisms involved are, however, largely unknown. The aim of this work is to review the impact of sexual hormones on the course of BD, regarding a clinical case.

**Objectives:**

To explore the role of sexual hormones in BD.

**Methods:**

Literature review using Medline database.

**Results:**

This is a case of a 36-year-old woman with type 1 BD who develops a manic episode after starting oral contraception (OC). This episode remitted with suspension of the pill. Estrogen and progesterone are involved in various aspects of brain function, such as brain development, synaptic plasticity, and modulation of neurotransmitter systems. Studies indicate that there is a relationship between ovarian hormones and intracellular signaling systems involved in the pathophysiology of BD. However, research on OC use in patients with mood disorders is limited. Recent studies state that OC aren’t associated with a worse clinical course and don’t negatively influence BD, while other studies show there is a subgroup of bipolar women that improve with hormonal stability, while others get worse.

**Conclusions:**

Further studies are needed to determine possible relationships between sexual hormones and BD, and it is essential to identify patients vulnerable to these risks by measuring baseline hormone levels, assessing hormone sensitivity through a history of mood changes during menstrual cycle and a history of previous mental health problems.

**Disclosure:**

No significant relationships.

